# Automated Coarse-Grained Mapping Algorithm for the Martini Force Field and Benchmarks for Membrane–Water Partitioning

**DOI:** 10.1021/acs.jctc.1c00322

**Published:** 2021-09-02

**Authors:** Thomas D. Potter, Elin L. Barrett, Mark A. Miller

**Affiliations:** † Department of Chemistry, Durham University, South Road, Durham DH1 3LE, United Kingdom; ‡ Unilever Safety and Environmental Assurance Centre, Colworth Science Park, Sharnbrook, Bedfordshire MK44 1LQ, United Kingdom

## Abstract

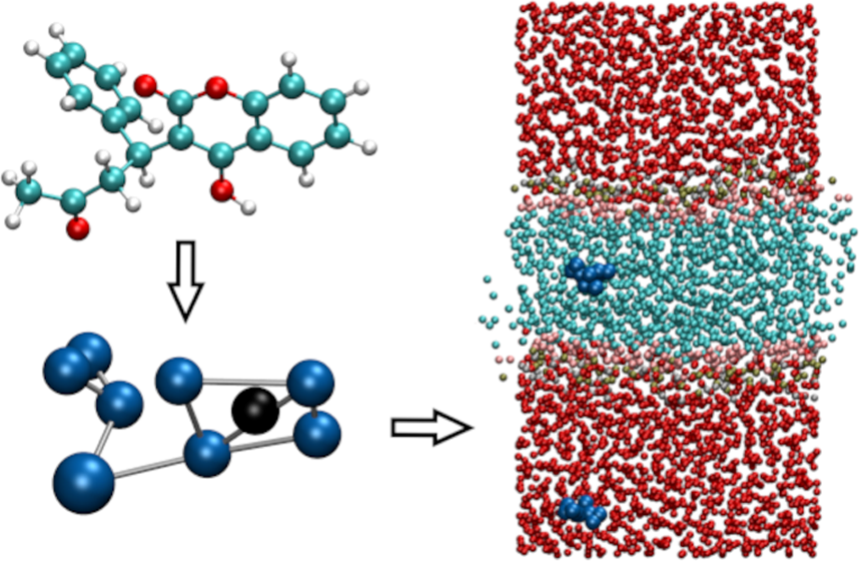

With a view to high-throughput simulations, we present an automated system for mapping and parameterizing organic molecules for use with the coarse-grained Martini force field. The method scales to larger molecules and a broader chemical space than existing schemes. The core of the mapping process is a graph-based analysis of the molecule’s bonding network, which has the advantages of being fast, general, and preserving symmetry. The parameterization process pays special attention to coarse-grained beads in aromatic rings. It also includes a method for building efficient and stable frameworks of constraints for molecules with structural rigidity. The performance of the method is tested on a diverse set of 87 neutral organic molecules and the ability of the resulting models to capture octanol–water and membrane–water partition coefficients. In the latter case, we introduce an adaptive method for extracting partition coefficients from free-energy profiles to take into account the interfacial region of the membrane. We also use the models to probe the response of membrane–water partitioning to the cholesterol content of the membrane.

## 1 Introduction

Computational screening is important in a variety of fields, from drug discovery^1^ to toxicology.^2^ The aim of screening is to narrow down a large chemical search space, thereby guiding time-consuming experimental testing toward formulations that are likely to have the desired behavior. Computational methods must generally be capable of high throughput to be viable for screening.

In the field of toxicity and environmental impact, one of the important physical quantities in screening is the membrane–water partition coefficient or its logarithm log *K*_MW_, which is a measure of the extent to which a molecule accumulates in biological tissues.^3−8^ Computational methods for predicting values of log *K*_MW_ vary widely in their sophistication and accuracy. Most simply, an approximate linear relationship between log *K*_MW_ and its counterpart for partitioning between *n*-octanol and water, log *K*_OW_, can be used and gives acceptable results for many molecules.^9^ Multiparameter linear free-energy relationships, based on the correlation of partitioning free energies with various molecular descriptors, have also been used to predict partitioning of solutes between different phases, including between lipid bilayers and water.^10−13^ The COSMOmic method, based on the COnductor-like Screening MOdel for Real Solvents (COSMO-RS),^14^ explicitly includes information about solute and membrane structures and compositions and so approximates how they interact with each other.^15^ However, in more complex cases, particularly the partitioning of ionic molecules, the inherent approximations in these methods can break down and more accurate methods are needed.^9^

Molecular dynamics simulations include a detailed description of the structure of the system, which is allowed to evolve under the equations of motion rather than relying on assumptions about how the different components affect each other. Molecular dynamics simulations have been used extensively to study the interactions of molecules with membranes,^16^ including the calculation of log *K*_MW_ directly from simulation trajectories.^17^ However, atomistic molecular dynamics simulations are computationally expensive, even when enhanced sampling techniques are used to accelerate the exploration of configurational space.^18,19^ Their use in high-throughput screening is therefore unfeasible.

A common strategy to improve the computational efficiency of simulations of biological and soft matter systems, including lipid bilayers, is the use of coarse-grained (CG) models. Coarse-graining involves removing degrees of freedom from an atomistic model by combining groups of atoms into single interaction sites. In such models, force calculations are cheaper because of the reduced number of sites. The reduction in the number of degrees of freedom also results in a smoother potential energy surface, allowing for longer simulation time steps and therefore faster exploration of configurational space than for a fully atomistic model.

There are two distinct stages to constructing a coarse-grained model: mapping and parameterization. Mapping is the division of the molecule into interaction sites, commonly known as beads. The mapping scheme sets the resolution of a coarse-grained model and thereby determines the speed-up that can be gained. There are many possible mapping schemes for any given molecule, even at a given level of bead resolution, and they vary in how well they capture properties of symmetry and structure.^20,21^ Mapping is often done manually but some methods have been developed to automate the process.^22,23^

Methods for the parameterization step are diverse in approach, and the models they produce have distinct strengths and weaknesses. Bottom-up methods fit the parameters of a coarse-grained model to a more detailed atomistic simulation and include structure-based methods,^24,25^ force matching,^26,27^ and relative entropy.^28^ It is possible to achieve an excellent representation of the atomistic system even with a much simpler model. However, some bottom-up methods suffer from poor transferability to conditions that are not represented in the atomistic reference and can be time-consuming to parameterize.^29−32^ Nevertheless, coarse-grained models involving more complex interaction types, such as local-density or volume-dependent potentials, have been shown to significantly improve transferability, often with only a small increase in computational cost.^33−36^ The transferability of simple pair-potential-based models can also be improved with appropriate parameterization schemes.^37,38^ There has also been progress in the use of machine learning methods to parameterize bottom-up coarse-grained force fields, including the automation of coarse-grained mapping.^39−41^ While this field is in its early stages, machine learning has the potential to further advance the efficiency and transferability of bottom-up coarse-graining.

Top-down models, in contrast, are fitted to macroscopic experimental thermodynamic data. Such models do not always reproduce fine structural details^32^ but are well suited to calculating thermodynamic properties and phase behavior.^42−44^ A number of top-down coarse-grained force fields have been developed.^45−48^ The most commonly used, particularly in biomolecular simulation, is Martini.^49,50^ Version 2 of Martini has been applied to a great variety of systems, including lipid bilayers,^51^ proteins,^52^ and polymers.^53,54^ Martini 2 consists of a number of predefined coarse-grained bead types, each of which is parameterized to represent specific types of chemical groups according to properties such as polarity and hydrophilicity.^49^ Each bead is intended to model around four heavy (nonhydrogen) atoms. The interactions between the beads are defined according to an interaction matrix, with interaction strengths parameterized to match partition coefficients of small molecules between organic solvents. Bonded parameters in Martini are often parameterized to match the underlying atomistic structure and can be generated in a bottom-up fashion from an all-atom simulation.^55^ The building-block approach of Martini gives the force field good chemical transferability at a given temperature. This feature is beneficial for high-throughput simulations, as it removes the need to fully reparameterize interactions for each system, as is the case in many other approaches.

The use of Martini in high-throughput screening requires a reliable, and preferably automated, way to generate models of organic solutes. The automartini method of Bereau and Kremer^22^ provides a way of automatically mapping and parameterizing small organic molecules within Martini. Automartini models for a set of 653 neutral organic molecules with up to 15 heavy atoms reproduced experimental octanol–water partitioning free energies^22^ with a correlation coefficient of 0.91 and a mean absolute error of 3.3 kJ mol^–1^. The method has also been used to carry out high-throughput studies of membrane interactions for extensive libraries of molecules^56^ and to investigate how well coarse-grained models cover chemical space.^57^ However, automartini’s methods for mapping and assignment of bonded interactions do have limitations. The mapping algorithm becomes impractically slow for molecules with more than about 25 heavy atoms due to the cost of enumerating all possible mapping schemes. The mappings are also not guaranteed to preserve the symmetry of the molecule. Additionally, models generated for extended ring compounds can require manual fine-tuning of the bonded parameters before they are suitable for use in simulations.^22^

In this paper, we present a method for generating coarse-grained Martini models that cover a wider chemical space than other methods. Our mapping approach is scalable to much larger molecules than previously possible, including those with complex ring structures. The models are consistently stable in molecular dynamics algorithms without reduction of the time step. This includes ring structures for which models with poorly chosen constraints would be unstable even at low time steps due to the practical numerical difficulty of simultaneously satisfying multiple, interdependent constraints. Our method also guarantees that the symmetry of the molecule is preserved during the coarse-grained mapping. We show that the resulting models reproduce experimental octanol–water and membrane–water partitioning data with good accuracy and devise an adaptive procedure for extracting the latter from the free-energy profile of a given solute. Collectively, these advances open up the use of Martini for high-throughput screening of thermodynamic properties.

The remainder of this article is organized as follows. Section 2 describes our automated scheme for mapping and parameterizing molecules for use with the Martini 2 force field. In Section 3, we test models from our scheme by using them to calculate octanol–water and membrane–water partition coefficients. Section 4 contains a discussion of the efficiency, strengths, and current limitations of our scheme. Finally, Section 5 provides a summary of the main advances presented in this article.

## 2 Methodology

**Figure 1:**
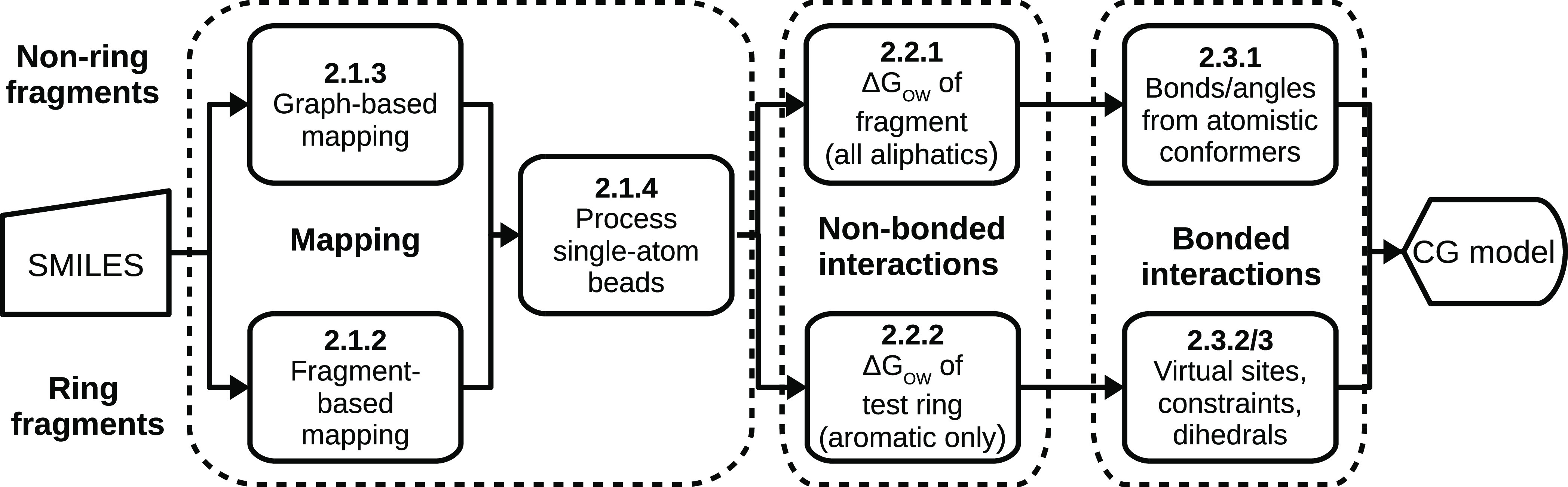
Flowchart outlining the three main stages of the algorithm: mapping of atoms onto beads and parameterization of nonbonded and bonded interactions. In the mapping and bonded interaction stages, the procedure differs for ring and nonring fragments, while for nonbonded interactions, the steps for aliphatic and aromatic fragments are distinct.

Our automated scheme for producing a coarse-grained model from a simplified molecular input line entry system (SMILES) code is summarized schematically in Figure 1. The three main stages of mapping and parameterization of nonbonded and bonded interactions are described in Sections 2.1–2.3 in turn. Each step in the procedure is labeled with the corresponding subsection number in the flowchart. A self-contained implementation of the full procedure is available to download with the Supporting Information. The code makes use of the open-source RDKit package,^58^ version 2020.09.1.

### 2.1 Mapping Scheme

#### 2.1.1 General Approach

Our three priorities for an automated mapping algorithm are that (1) the method must generate mapping schemes compatible with the Martini framework, (2) the mappings must respect the symmetry of the molecule as much as possible, and (3) the algorithm must readily scale to large molecules.

Our approach, which meets all of these criteria, consists of three steps. First, we isolate ring systems within the molecule and map them using a fragment-matching algorithm. Next, we apply a version of the graph-based method of Webb et al.^23^ to systematically map nonring fragments of the molecule. Finally, we apply a postprocessing step to deal with any individual atoms that could not be mapped in the previous two steps. Each of these stages of the algorithm is described in the rest of this section.

#### 2.1.2 Ring Structures

Within the Martini framework, ring compounds are typically modeled by small (S) beads, which represent 2–3 heavy atoms, joined together in a rigid structure by a combination of LINCS constraints and virtual sites.^49,52,59^ The structure of such representations makes simulations more prone to instabilities at longer time steps because of the difficulty in satisfying a large number of constraints simultaneously. Hence, it is harder to determine a robust and practical mapping scheme for rigid molecules than for flexible molecules.

**Figure 2:**
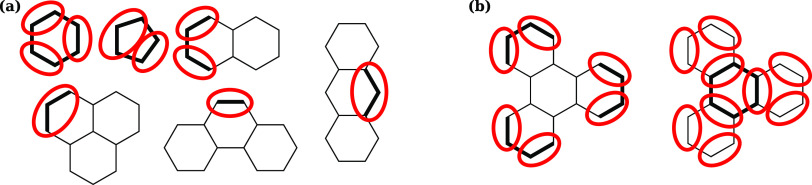
(a) Predefined mappings for rings with examples where these fragments occur. The fragments involved are in bold, with red ellipses indicating the coarse-grained beads. (b) The iterative mapping process for a triphenylene ring fragment.

Our automated framework for coarse-grained mapping of ring compounds is designed to deal with fragments containing combinations of fused five- and six-membered rings and is illustrated in Figure 2. There are two steps: grouping of the outer parts of the ring system according to predefined patterns, followed by mapping of the remaining inner fragments using graph-based grouping.

The outer parts are mapped by finding continuous chains of atoms that are part of only one ring and mapping them, as shown in Figure 2. These mapped fragments are removed, and the process is repeated for the remaining fragments. If none of the unmapped fragments forms a ring, then the remainder of the ring system is mapped by applying one iteration of the graph-based mapping system described in Section 2.1.3 but with a maximum path length of two bonds.

#### 2.1.3 Graph-Based Grouping

**Figure 3:**
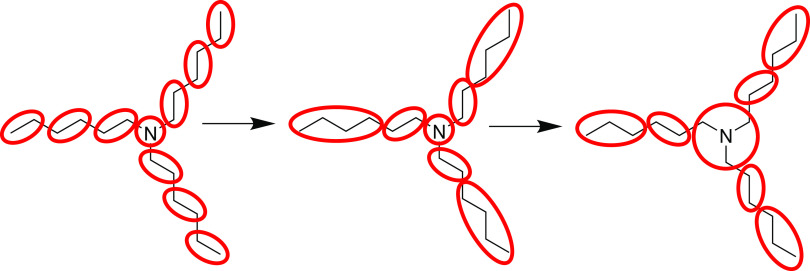
Mapping scheme for trihexylamine, including two iterations of graph-based grouping followed by postprocessing to remove a single-atom bead.

The graph-based mapping method developed by Webb et al. uses only information about the connectivity of the atoms.^23^ It is based on representing a molecular structure as a graph in which atoms are the nodes and bonds are the edges. The nodes are ranked and then combined according to some metric. This ranking may be done in a number of ways, and we have adapted the spectral ordering method.^23^ In this approach, the whole molecule is analyzed at once, with symmetrically equivalent fragments treated simultaneously, ensuring that the symmetry of the molecule is automatically respected. The whole mapping process for an example nonring compound is shown in Figure 3, including the postprocessing step described in Section 2.1.4.

In spectral mapping, the molecule is represented by an adjacency matrix **A**. A_*ij*_ is 1 if nodes *i* and *j* are bonded and 0 otherwise. The diagonal terms are used to weight the nodes. The weighting of node *i* in our scheme is given by
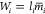
1where *l*_*i*_ is the maximum number of bonds between any two heavy atoms in the bead and *m̅*_*i*_ is the mean atomic mass of all of the nonhydrogen atoms within the bead. Using *l*_*i*_ in the definition of weight favors the formation of beads with branched internal structures compared to long, linear beads, while the inclusion of *m̅*_*i*_ is a tie-breaker for chemically distinct beads with the same branching structure. Other weighting functions are possible, but this simple product reliably generates Martini-compatible mappings even for highly branched structures. The nodes are then ranked according to their centrality scores,^23^ which are obtained by diagonalization of 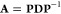
2where **P** contains the eigenvectors of **A**, and **D** is a diagonal matrix of its eigenvalues. The centrality scores of the nodes in the graph are the components of the eigenvector corresponding to the largest eigenvalue. Starting from the lowest-ranked node, the nodes are grouped with their neighbors. For each node *i*, all neighbors with equal or higher rank are identified and, of those, the one with the closest ranking is grouped with node *i*. If multiple nodes of the same rank simultaneously qualify to be combined with the same neighbor, all of these nodes are grouped together. At this stage, all ring fragments generated by the procedure in Section 2.1.2 are excluded. If there is no suitable neighbor, node *i* remains as its own group.

We have introduced an additional constraint on bead size for compatibility with Martini. For each candidate node *i*, the maximum path length, *l*_*i*_, between any two heavy atoms within the bead is measured. If *l*_*i*_ is greater than three bonds, the combination step is rejected and the nodes making up the candidate node remain as their own groups. This ensures that no group grows too large to model using Martini while still allowing smaller nodes to combine in other parts of the molecule.

This procedure can be carried out iteratively until the desired level of coarse-graining is achieved. In practice, we iterate until no new beads are formed.

#### 2.1.4 Postprocessing

In some cases, the mapping scheme results in single atoms that cannot combine with others without exceeding the size limit. There is no appropriate Martini representation for a single heavy atom, so a postprocessing step is needed to take care of these cases. The nodes are ranked by centrality score as before, and the highest-ranked neighbors of any single-atom nodes are identified, excluding any ring beads. From each of these neighbors, one heavy atom is transferred to the bead of the single atom. If there are multiple neighbors with the same rank, then a heavy atom is removed from each, ensuring that the symmetry of the molecule is retained. If this results in new single-atom beads, the procedure is repeated until all beads have *l* between 1 and 3. An example of a molecule for which this process is necessary is given in Figure 3.

In some cases, this process can fail to terminate because single atoms are passed back and forth between beads. Two constraints are placed on the procedure to prevent this. First, nodes containing atoms that were either single-atom nodes or bonded to a past single-atom node in previous iterations, are tagged. If a tagged node is the highest-ranked neighbor of a single-atom node, then it is passed over and an atom is taken from the next highest neighbor. Second, in cases where the single-atom node is bonded to a terminal node with path length *l* = 1, the single-atom node and the terminal node are combined to form a new bead. This prevents the formation of terminal single-atom nodes that have no way to combine with any other atoms.

Finally, if the only appropriate neighbor for a single-atom node is a ring node, the two nodes are combined, effectively absorbing the lone atom into the mapping for the ring. In cases where there is a choice between two ring beads, the one with the highest centrality score is chosen.

### 2.2 Nonbonded Interactions

#### 2.2.1 Aliphatic Fragments

Parameterization of nonbonded interactions in the Martini framework consists of selecting from the list of predefined bead types that make up the force field. For parameterizing the subset of beads that represent nonaromatic fragments, we have adopted the bead-selection approach from the automartini method of Bereau and Kremer, which is based on matching to octanol–water partitioning free energies (Δ*G*_OW_).^22^

In this procedure, once a mapping scheme has been decided, Δ*G*_OW_ is predicted for each of the neutral fragments. The prediction is preferentially done using ALOGPS, a neural network trained to predict octanol–water partitioning; it has been shown to produce high-quality results for a diverse range of organic molecules.^60^ In cases where ALOGPS is not applicable (e.g., for wholly inorganic fragments such as NO_2_), the Wildman–Crippen approach is used; this method determines Δ*G*_OW_ from contributions by the atoms that make up the fragment.^61^ The Martini bead type with the closest Δ*G*_OW_ to the ALOGPS or Wildmann–Crippen value is chosen to represent that fragment.

In our implementation of this part of the algorithm, we have obtained Δ*G*_OW_ from ALOGPS for all organic fragments that fit the bead size limits described in Section 2.1.3 and included these values in a data file along with the code (see the Supporting Information (SI)). This self-contained implementation eliminates dependence on the web server hosting the neural network.

Some bead parameterizations also make use of hydrogen-bonding properties. Neutral beads with a predicted Δ*G*_OW_ within ±1.0 kJ mol^–1^ of the Nda (nonpolar acceptor and donor) bead type can be assigned as either Na, Nd, or Nda beads if they include hydrogen-bond acceptors, donors, or both. The hydrogen-bonding properties of the atoms are determined using the RDKit library’s chemical feature module. Otherwise, the bead type is selected based on Δ*G*_OW_, as described above. Bead types for charged fragments are chosen purely based on hydrogen-bonding properties. Fragments can be assigned as Qa, Qd, or Qda as described above, or as Q0 if no hydrogen-bonding atoms are present.

#### 2.2.2 Aromatic Fragments

**Figure 4:**
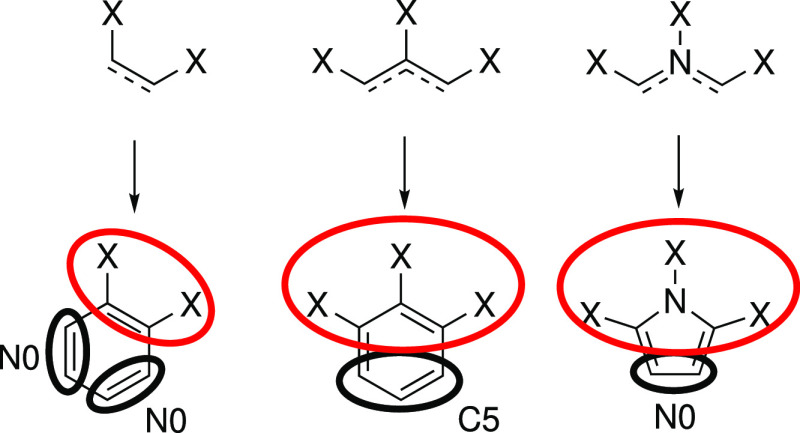
Procedure for constructing full rings from mapped ring fragments.

Fragments that are part of nonaromatic rings are parameterized in the same way as nonring fragments. For aromatic rings, we have developed a different approach, designed to take account of heteroaromatic fragments and variable-resolution ring mappings (i.e., two or three ring atoms, plus their substituents, per bead). This is illustrated in Figure 4.

Aromatic fragments are detected automatically using the aromaticity model in RDKit and are recognized in the fragment’s SMILES code. For each aromatic fragment arising from the mapping algorithm, we construct a standardized test molecule containing the aromatic fragment along with the rest of a six-membered ring. In cases where this does not result in a valid structure (most commonly with nitrogen-containing heteroaromatics), we instead form a five-membered ring. The bead type is then chosen in a similar way to nonring fragments, except that the Δ*G*_OW_ of the whole ring is matched to that of a Martini ring or dimer (depending on the CG resolution). The added carbon atoms are represented by predetermined bead types, which were chosen by finding the closest Δ*G*_OW_ match for benzene (either an N0 trimer or a C5 dimer). Note that these bead selections do not match the types used in most existing Martini models, but we chose the bead selections that fitted best with Δ*G*_OW_ for consistency with the approach used for aliphatic fragments. A similar observation was made in a recent paper by Kanekal and Bereau.^57^

### 2.3 Bonded Interactions

#### 2.3.1 Bond Lengths and Angles

Bond lengths and angles are modeled using harmonic potentials. We parameterize these contributions using atomistic conformers of the molecule, generated using the experimental-torsion knowledge distance geometry (ETKDG) method,^62^ which is based on information from experimental crystal structures, followed by minimization using the universal force field (UFF).^63^ We use 200 such conformers for the parameterization to provide a balance between computational cost and convergence, as discussed in Section 4.1. The set of conformers is mapped to a CG resolution, and bond lengths and angles are taken as the average values across this set. The standard values from the Martini model are used for force constants. Further details on the effect of different bond parameterization schemes can be found in the SI.

The CG beads involved in bond and angle interactions are simply groups of two or three fragments that are bonded together in the atomistic representation of the molecule. This does not apply to pairs or triplets in which all of the beads are part of the same ring. Bonded interactions within rings are modeled using a network of constraints, dihedrals, and virtual sites, as described in the following sections.

#### 2.3.2 Virtual Sites

Simulations of coarse-grained ring structures often suffer from instabilities at longer time steps because of the complex networks of constraints that must be simultaneously satisfied to enforce the correct molecular geometry. These convergence problems can be mitigated by representing some of the beads in aromatic systems as virtual sites. The virtual sites exert forces on other sites as normal, but any force acting on them is redistributed to the surrounding beads.^64^ The position of a virtual site is defined as a weighted combination of the positions of other beads, which also defines how the force is redistributed. Because virtual sites do not interact through normal bonded interactions, they reduce the complexity of the bonded structure of CG ring compounds, improving their stability in simulations. This approach has been successfully deployed, for example, in the treatment of sterols by Melo et al.^59^

We have introduced an automated procedure to include virtual sites in coarse-grained models of large ring systems. Fragments are split into outer and inner sets of beads, which are designated real and virtual sites, respectively. The first stage of this process is to define a two-dimensional coordinate system for each ring system. The axes of this system are found by diagonalization of the inertia tensor, **I**, of the ring. The eigenvectors associated with the two smallest eigenvalues of **I** define an orthogonal basis in the plane of the ring. The coordinates of the ring system are then projected onto this two-dimensional coordinate set. The outer beads are determined by finding the convex hull of the ring system,^65^ and these are designated as real sites. The remaining ring beads are designated as virtual sites. Any virtual site that is bonded to a nonring substituent is moved to the list of real sites.

There are many methods for handling virtual sites within simulations. We use a construction in which the position of each virtual site, **v**, is described by a linear combination of its four nearest neighbors within the same ring system (or three if the system has only three real sites)
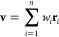
3where *w*_*i*_ and **r**_*i*_ are the weighting and position of site *i*, respectively, and *n* is the number of sites used to express **v**. Choosing appropriate weights for a set of real and virtual sites requires finding a solution to eq 3 for a representative configuration of the real and virtual beads. By definition, rigid fragments have little internal variation between conformers, so any of the conformers generated in Section 2.3.1 can be used. When *n* = 3 or 4, there are infinitely many solutions of eq 3. However, in practice, we would like the solution that gives the most balanced distribution of forces across the constructing sites. This is done with the aid of simple geometric constructions, as described below.

**Figure 5:**
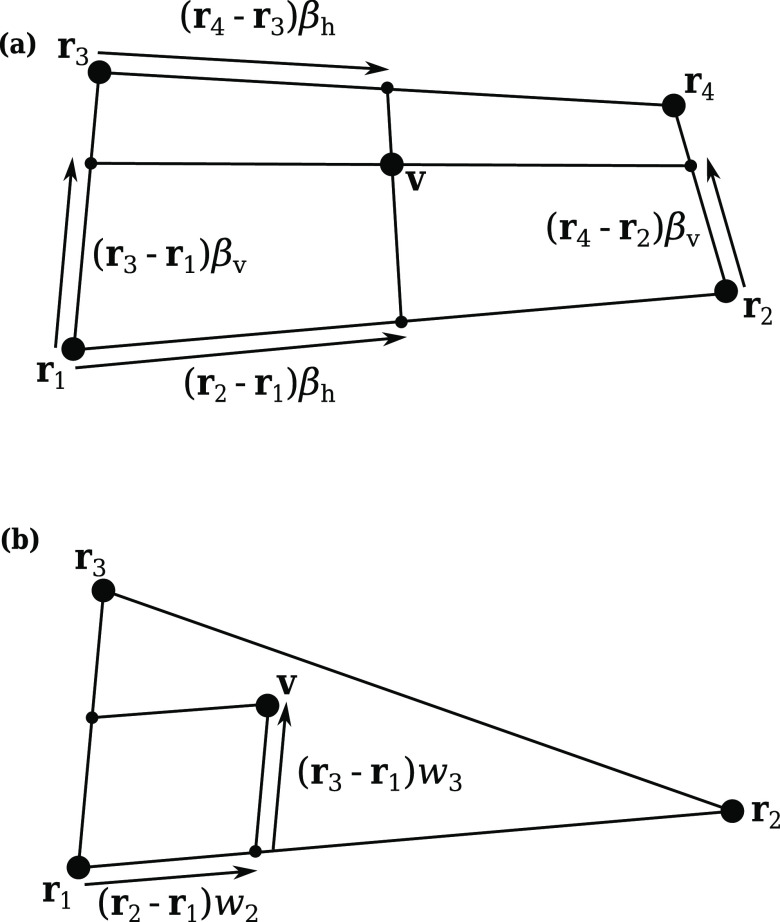
Schematic of the geometric constructions used to determine weights for virtual sites when (a) *n* = 4 and (b) *n* = 3.

When *n* = 4, there is no unique solution to eq 3. We can choose weights that balance the force redistributed onto each neighbor by placing the virtual site at the intersection of two lines crossing the quadrilateral. The ends of the two lines are defined by the parameters β_h_ and β_v_, which represent fractions of the edges of a quadrilateral, as shown in Figure 5a. The weights are given geometrically by
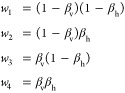
4After shifting the coordinates so that **r**_1_ is at the origin, β_v_ can be determined by solving the quadratic equation (see the SI for a full derivation)
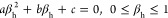
5where
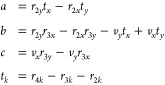
6β_v_ is then calculated from
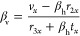
7

When *n* = 3, as shown in Figure 5b, the procedure is simpler. Again shifting **r**_1_ to the origin, *w*_2_ and *w*_3_ can be calculated by solving
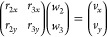
8The weight on **r**_1_ is then calculated from
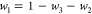
9

#### 2.3.3 Ring Geometry

The network of constraints and dihedrals in a ring compound is designed to enforce a rigid geometry where required while still allowing for large simulation time steps. We therefore aim to generate a model with the minimum number of interactions necessary to keep the correct molecular geometry based on a hinge construction.^59,66^ First, the real sites are connected by a closed ring of distance constraints,^67^ giving an outer frame. Further constraints are then placed in a zig-zag pattern across the ring system, splitting it into triangles and creating a series of hinges. Each of these hinges is kept at the correct angle using a single dihedral. The dihedrals are placed to minimize the sharing edges. Pseudocode for the algorithm for adding constraints and dihedrals is included in the SI, along with examples of the results for various ring structures and a discussion on the limits of the dihedral generation algorithm.

**Figure 6:**
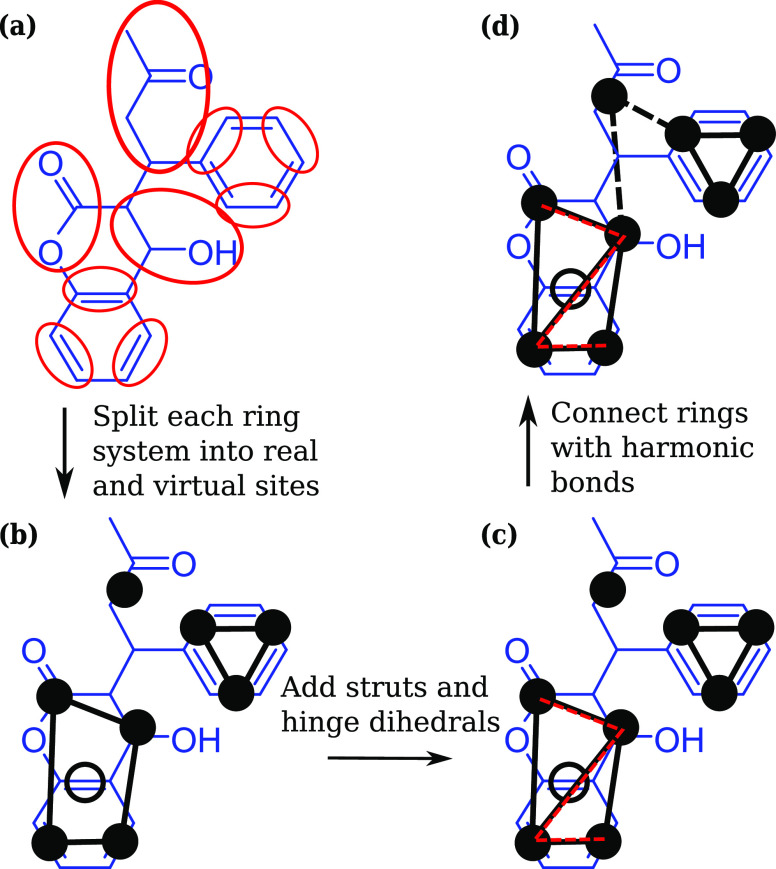
Schematic illustration of the process for generating virtual sites and ring constraints for warfarin. (a) Mapping of atoms onto coarse-grained beads (red ellipses), (b) ring constraints (thick lines), real sites (filled circles), and a virtual site (open circle), (c) dihedral constraint for the double ring (dashed red line), and (d) bonds connecting the ring fragments (thick dashed lines).

The process for generating ring structures, including virtual sites, constraints, and dihedrals, is illustrated in Figure 6 for a warfarin molecule. This molecule includes multiple ring systems that are treated separately from each other by the algorithm before being joined to make the molecule.

## 3 Partition Coefficients

To test the method presented in Section 2, we use it to derive coarse-grained models for a set of 87 molecules. We benchmark the models by evaluating octanol–water and membrane–water partition coefficients using simulations and comparing them with experimentally measured values.

### 3.1 General Simulation Parameters

Coarse-grained simulations were carried out using Gromacs 2018.2.^68^ The *new-rf* set of molecular dynamics parameters were used; these were proposed by de Jong et al. as providing the optimal balance between accuracy and computational efficiency.^69^ A leapfrog integrator was used, with a time step of 20 fs. Electrostatic interactions were handled using the reaction field method,^70^ with a relative permittivity of 15.0. Cutoffs for the van der Waals and electrostatic interactions were set to 1.1 nm. Temperatures were kept constant at 303 K using the velocity rescale method^71^ with a coupling constant of 1.0 ps. The Parrinello–Rahman barostat^72^ with a coupling constant of 12.0 ps and a reference pressure of 1 bar was used in constant NPT simulations.

Coarse-grained phospholipid parameters were taken from the library of lipids provided on the Martini website.^51^ Cholesterol was modeled using the virtual site representation parameterized by Melo et al.^59^ Water and ions were modeled using the standard Martini parameters, in which one bead represents four water molecules. Ten percent of the water molecules are the so-called antifreeze particles and prevent the unphysical freezing of water at ambient temperatures.^49^

### 3.2 Octanol–Water Partitioning

We start by testing the ability of models generated using our method to reproduce octanol–water partition coefficients (log *K*_OW_). Initial structures for these calculations were created by randomly placing 400 water beads or octanol molecules into a cubic box, using the gmx insert-molecule tool included with Gromacs. This was followed by energy minimization and a 10 ns constant NPT run to generate an equilibrated box of solvent.

Solvation free energies in octanol (Δ*G*_O_^solv^) and water (Δ*G*_W_^solv^) were calculated using the Bennett acceptance ratio (BAR) method.^73^ A solute molecule was placed at a random position in a box of solvent. A series of simulations were carried out in which the interactions between the solute and the solvent were turned off, as defined by a scaling parameter λ. Soft-core potentials were used to prevent singularities when λ is close to 0. The octanol–water partitioning free energy was then calculated directly from
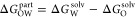
10giving the partition coefficient
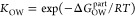
11

**Figure 7:**
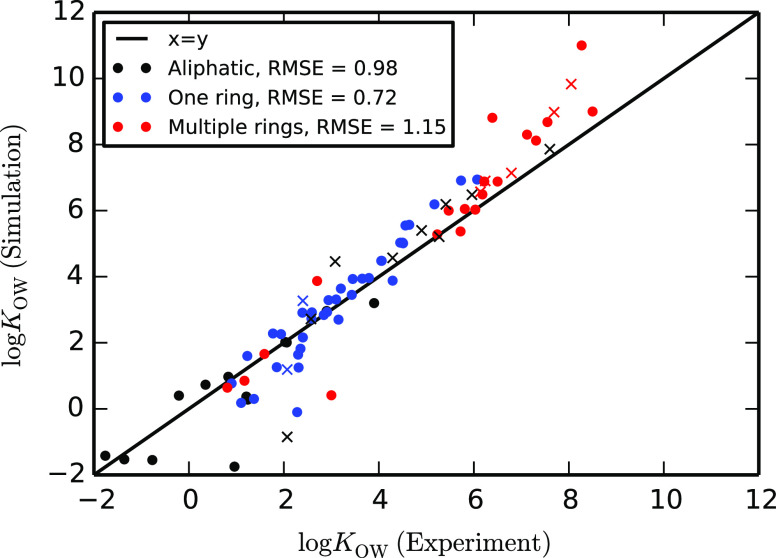
Octanol–water partition coefficients (log *K*_OW_) for a set of organic molecules using coarse-grained models compared to experiment (dot markers) or predicted ALOGPS values (cross markers) where experimental data are not available.

Our test set for log *K*_OW_ is a diverse set of organic molecules, including a variety of functional groups, alkyl chain lengths, and ring structures. A full list is given in the SI. Figure 7 shows the simulation results against experimental measurements. For 16 of the 87 molecules, experimental values were not available, and log *K*_OW_ predictions from the ALOGPS server were used instead.^60^

Overall, there is a good agreement between the experimental and simulated values, although there are some distinct outliers. The most hydrophilic molecules show more scattered data. Their behavior tends to be dominated by hydrogen-bonding and other directional interactions that are not explicitly included in Martini. These molecules could benefit from the polarizable Martini model^74^ or from the recently published Martini 3 force field,^75^ and future work will focus on extending the approach to these force fields. There is also a small, systematic overestimation of log *K*_OW_ in the more lipophilic ring compounds. These molecules are almost exclusively multihalogenated aromatics, especially those where a dichloro group is mapped to a single bead.

**Figure 8:**
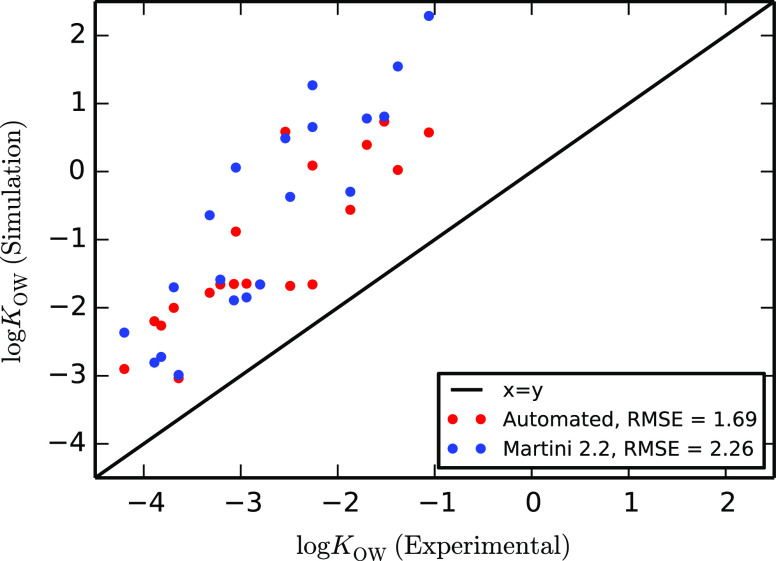
Octanol–water partition coefficients (log *K*_OW_) for amino acids calculated using Martini 2.2 and automated models compared to experimental values.

We also tested our method against a set of existing models from the Martini force field, namely, amino acids. We have compared models for all 20 amino acids generated using our method with those from the Martini 2.2 library.^52^ Octanol–water partitioning data for those models are compared to experimental values^76^ in Figure 8. Both sets of models systematically overestimate log *K*_OW_, but our automated models (root-mean-square error in log *K*_OW_, RMSE = 1.69) outperform the standard Martini 2.2 models (RMSE = 2.26). Of the 20 solutes, 14 have improved results with the automated models. The systematic shift in both sets can be traced to the limitations of the Martini 2 model. It has been shown that using the polarizable Martini model significantly improves the partitioning behavior of highly polar molecules like amino acids, but this is outside the scope of the present study.^74,77^ However, it is still encouraging that an automated approach to parameterization results in some improvement over handmade coarse-grained models within the limitations of the force field itself for this set of compounds.

### 3.3 Membrane–Water Partitioning

We now test the ability of our automatically generated coarse-grained models to predict membrane–water partition coefficients (log *K*_MW_) as a step toward high-throughput screening of organic molecules for bioaccumulation.

#### 3.3.1 Calculating Partition Coefficients

Initial coarse-grained lipid structures were generated using the insane.py tool, which constructs a solvated bilayer by placing lipids and water beads on a cubic lattice with the *z-*axis normal to the bilayer.^51^ All bilayers in this study consist of 128 lipid molecules (two leaflets of 8^2^ lipids being a convenient initial construction). A number of water beads were replaced by an equal number of cations and anions to give a 150 mM salt concentration. To facilitate comparison with the experimental data available for each solute, the simulations used either a palmitoyloleoylphosphatidylcholine (POPC) or a dimyristoylphosphatidylcholine (DMPC) membrane on a case-by-case basis. Our simulations predict that the differences between log *K*_POPC–W_ and log *K*_DMPC–W_ are small but do vary in magnitude between the solutes. For example, in the case of *nnn*-trihexylamine, the mean value across 10 replicas with each membrane was 6.21 for a POPC bilayer and 5.80 for DMPC. For warfarin, the two values were 2.79 and 2.10.

Energy minimization was carried out on the initial structures, followed by a 100 ns constant NPT molecular dynamics run. The average *x* and *z* box lengths were calculated from this run, and a single structure with this size was extracted from the trajectory to be used as a starting bilayer configuration.

In this study, umbrella sampling was used to generate probability distributions of solutes across membranes. For each membrane/solute combination, a series of umbrella simulations were run with the distance along the *z*-axis between the centers of mass of the membrane and the solute restrained at values between 0.0 and 5.0 nm, separated by 0.1 nm. The restraint was achieved using a harmonic potential with a force constant of 1000 kJ mol^–1^ nm^–2^. The distance was defined using the cylinder method, in which the center of mass of the membrane is calculated using only the lipid molecules that lie within a cylinder of radius 1.5 nm, whose axis is parallel to *z* and passes through the center of mass of the solute. This reduces the effect of any undulations of the lipid bilayer.^78^ Each simulation included two solute molecules being sampled separately across a different leaflet of the membrane to improve sampling without increasing simulation time.^79^

For each umbrella window, the two solute molecules were inserted into the equilibrated bilayer at a fixed *z*-coordinate and random *x* and *y* coordinates. A steepest-descent energy minimization was carried out, followed by a 100 ps equilibration simulation with a 2 fs time step. A 50 ns simulation with a 20 fs time step was then carried out. The first 1 ns of this run was treated as additional equilibration, and the remaining 49 ns were used for analysis. A probability profile was generated using the weighted histogram analysis method (WHAM).^80^

The membrane–water partition coefficient is defined by
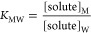
12where [solute]_X_ is the concentration of the solute in the membrane (X = M) or in the aqueous phase (X = W). Extracting *K*_MW_ from the probability profile requires the profile to be split into parts associated with the solution and with the bilayer. This is complicated by the fact that the interface region is large in relation to the size of the membrane and often has a significantly different interaction with solutes than either the membrane or water. Previous studies have tackled this issue by scaling the contribution to the membrane probability in the interfacial region.^17^ Another approach is simply to use a fixed cutoff for a particular lipid type. Both methods work well for most molecules but can neglect important features of the profile when a solute interacts strongly with the interfacial region.

Our approach is based on the assumption that any deviations from the probability in the bulk water region must be due to interactions with the membrane, including the interfacial region. We therefore define a hard cutoff between the two regions but with an adjustable position that depends on the point at which the free-energy profile of the solute starts to deviate from the pure water value. Using this cutoff, we can calculate *K*_MW_ using
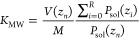
13Here, the membrane–water system has been divided into notional layers parallel to the *xy* plane, and *z*_*i*_ refers to the position of layer *i*, running from *i* = 0 at the center of the membrane to *i* = *n* in the outermost water layer (in this work, we have used a resolution of 100 layers). *R* is the index of the outermost layer designated as part of the membrane region for a given solute. *P*_sol_(*z*_*i*_) is the probability of finding a given solute molecule in layer *i. V*(*z*_*n*_) is the volume of the outermost water layer and *M* is the mass of one leaflet of the lipid bilayer. This gives *K*_MW_ in units of dm^3^ kg^–1^, in common with many experimental studies. The deviation from bulk behavior at a layer *j* is estimated using the root-mean-square deviation (RMSD) of the free-energy profile between *z*_*n*_ and *z*_*j*_. By iterating over *j* between *j* = *n* and 0 and calculating the RMSD at each point, we can define the cutoff as the layer *j* = *R*, at which the RMSD first increases above 0.1.

**Figure 9:**
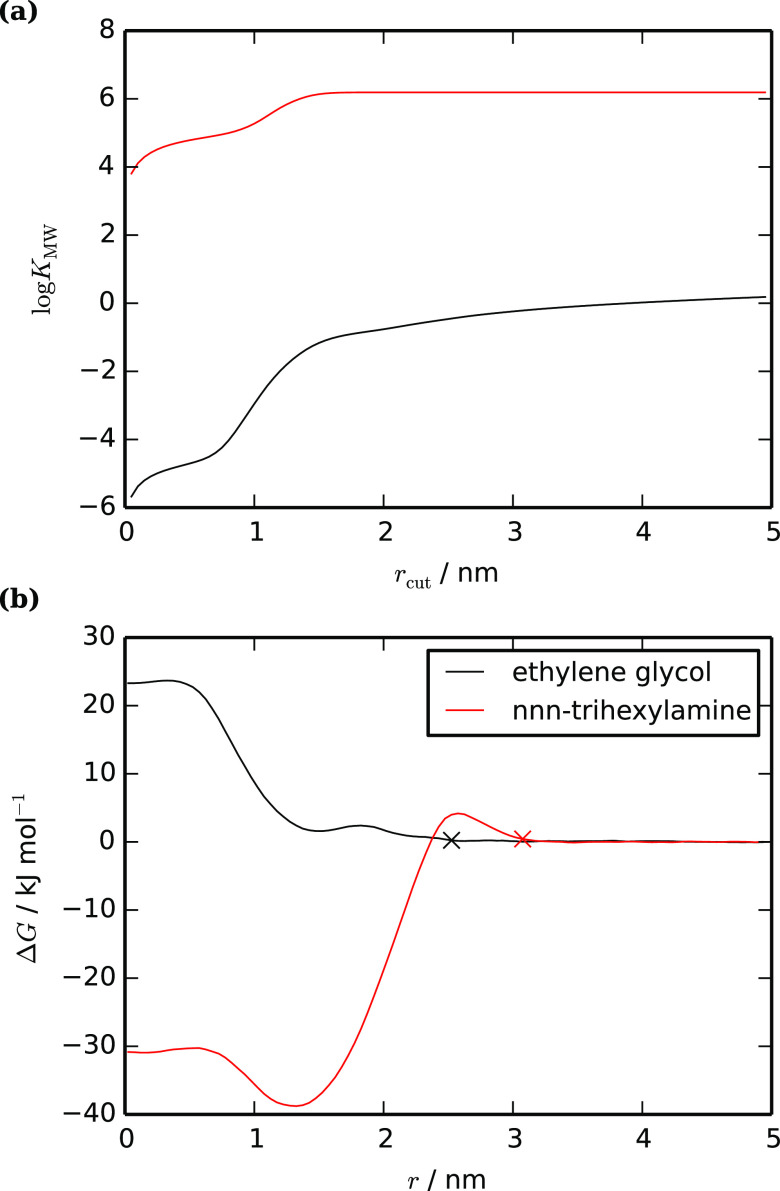
(a) Convergence of log *K*_MW_ with cutoff between the membrane and water for ethylene glycol and trihexylamine. (b) Free-energy profiles for the two compounds, with cutoff from an RMSD threshold of 0.1 indicated by crosses. The center of the membrane lies at *r* = 0.

The specific value for the RMSD threshold used here is arbitrary, but we have found in most cases that the results are insensitive to the threshold applied. This is because, in cases where the interface is an important contributor to the membrane partitioning, the change in free energy on approaching the interface from the water phase is usually quite sharp. With the exception of very hydrophilic compounds, the calculated *K*_MW_ does not significantly depend on the way the probability distribution is split between phases (fixed or variable hard cutoff or scaled cutoff). This is illustrated in Figure 9 for trihexylamine and ethylene glycol. For the former, log *K*_MW_ is dominated by the well inside the membrane, so the position of the cutoff does not affect the result once the whole tail region is covered. In the case of ethylene glycol, there is only a shallow, local free-energy minimum inside the membrane, making the interfacial region more influential on partitioning, and log *K*_MW_ does not fully converge with cutoff distance. However, this merely reflects the fact that the free-energy density for this solute is lower in water than anywhere in the membrane, so that artificially associating more water with the membrane will always appear to increase *K*_MW_. Nevertheless, as indicated in Figure 9b, using an RMSD threshold of 0.1 does lead to a sensible division between the membrane and water. Overall, for these very hydrophilic compounds, getting a precise estimate of log *K*_MW_ is challenging, but, by its very nature, this problem only arises when log *K*_MW_ is very small.

As a spot-check on the statistical uncertainty of the log *K*_MW_ values calculated by our protocols, we repeated the umbrella sampling procedure 10 times for each of four contrasting molecules using different randomly inserted coordinates of the solute for the starting coordinates. The standard deviation across the 10 calculated values of log *K*_MW_ is an estimate of the uncertainty in any one of them (i.e., the typical error present without the repeats). The results from these simulations are shown in Table 1. The estimated uncertainties are all below 0.2 log units. Ethylene glycol has the largest uncertainty, which is due to the lack of a clear-cut division between the membrane and water regions for hydrophilic molecules, as explained above. Experimental measurements of log *K*_MW_ are not always published with error estimates, but the statistics given in one recent study^8^ (using solid-supported lipid membranes) imply that the uncertainty in an individual measurement was in the range of ±0.03 to ±0.2 with a mean of ±0.13. This level of statistical uncertainty is very similar to that of the spot-checks listed in Table 1.

**Table 1:** Mean and Standard Deviation (SD) of log *K*_MW_ Calculated from 10 Separate Replicas

solute	mean	SD
trihexylamine	6.21	0.12
warfarin	2.79	0.12
glycerol	0.56	0.04
ethylene glycol	–0.23	0.18

**Figure 10:**
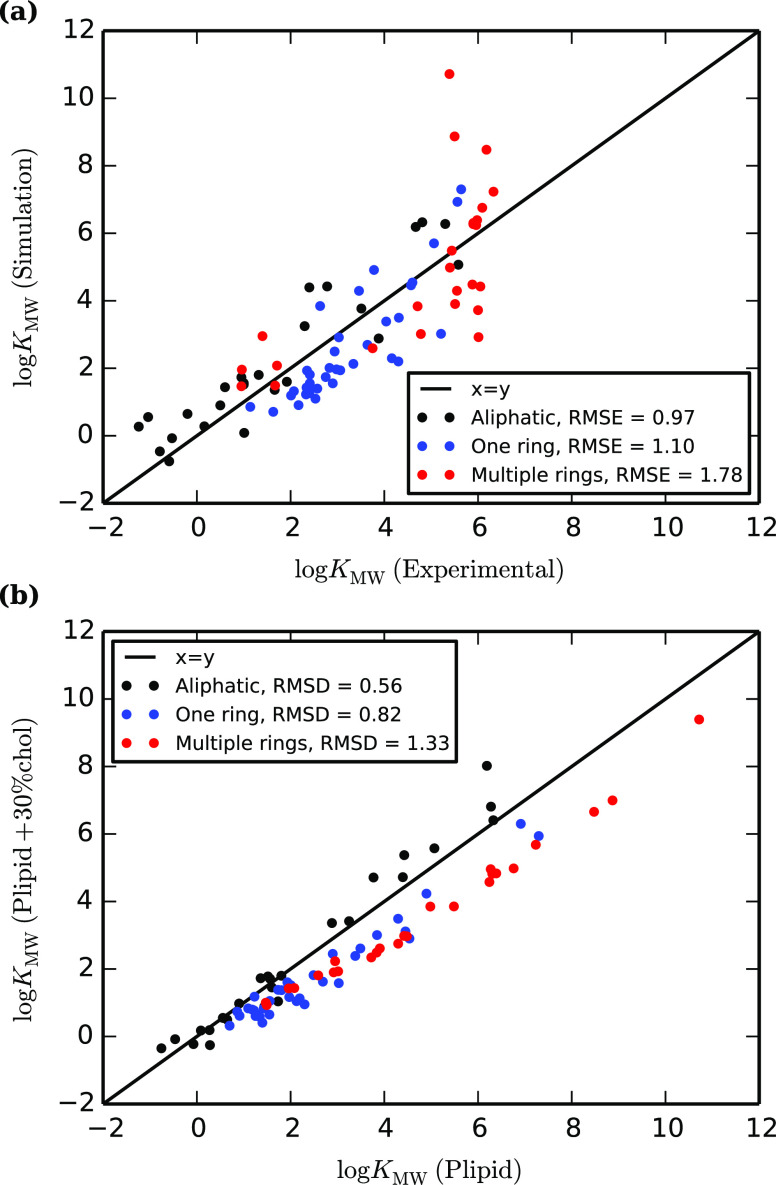
Membrane–water partition coefficients (log *K*_MW_) for a range of small organic molecules using coarse-grained models. (a) Coarse-grained vs experimental in a pure phospholipid membrane and (b) log *K*_MW_ in a phospholipid + 30% cholesterol vs pure phospholipid membrane, both from the coarse-grained models.

Figure 10a shows log *K*_MW_ values for our test set from CG simulations compared to experimental values.^3,7,8,81−95^ For aliphatic compounds, the agreement between the simulation and experiment is essentially the same as for octanol–water partitioning (RMSEs 0.97 and 0.98, respectively), despite the fact that membrane–water partitioning is more challenging both experimentally and computationally. Single-ring compounds also perform well. The least satisfactory results are for a set of halogenated biphenyl compounds. The trend in simulated log *K*_MW_ for this set is similar to that in both the simulated and experimental log *K*_OW_, but the experimental log *K*_MW_ data have a very small spread. It is therefore not clear whether the discrepancy should be attributed to the models, the experimental data, or a combination of the two. The fact that the octanol–water trend is correct suggests that the models themselves are basically sound, but there may be subtleties in the interactions between halogenated fragments and the membrane, which are not captured well by the bead types in Martini 2.

#### 3.3.2 Membrane Composition

The partitioning of molecules into membranes with realistic compositions is important for the prediction of how compounds interact with real biological systems. A number of experimental and simulation studies have shown that the presence of cholesterol in a lipid bilayer has a significant impact on partitioning.^96−98^ Cholesterol is known to have an ordering effect on bilayer systems,^99^ and this change is hypothesized to reduce the permeability of the membrane.^96,100−102^ However, most studies to date have focused only on small sets of molecules. Automated coarse-graining could be valuable in exploring in depth how different families of molecules interact with different membrane compositions.

**Figure 11:**
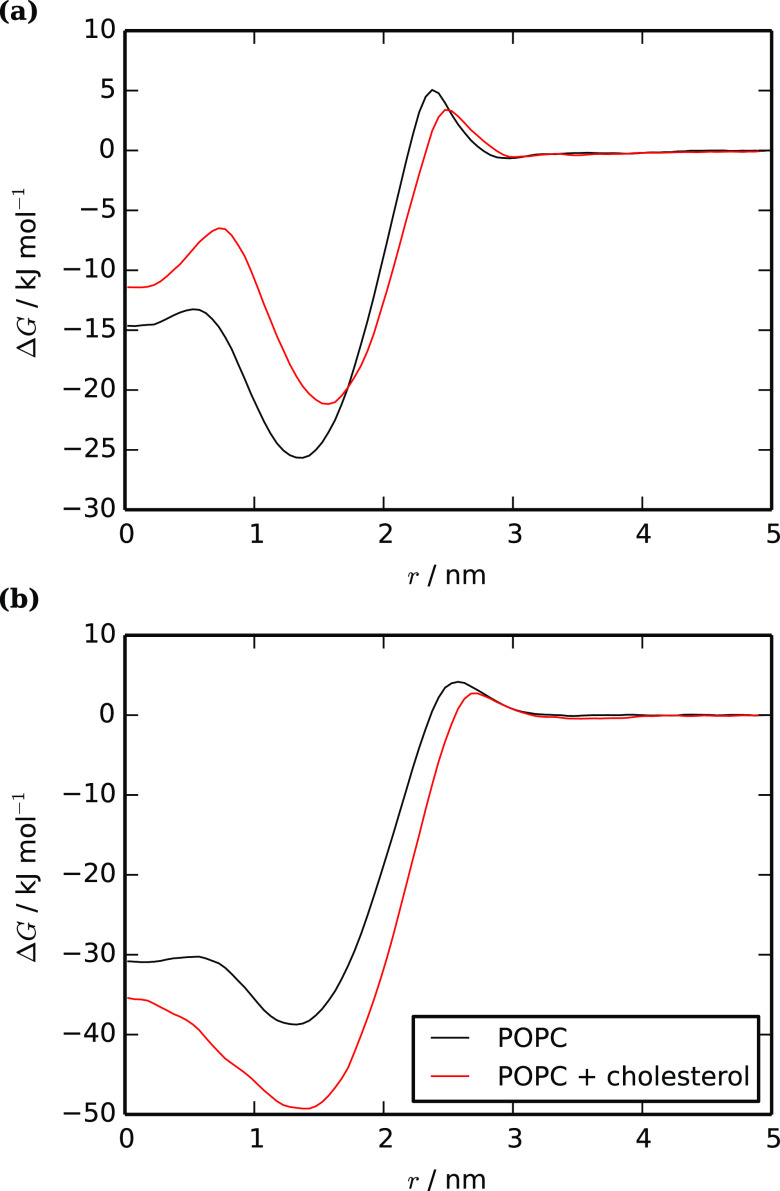
Potentials of mean force between the bilayer center (*r* = 0.0) and bulk water (*r* = 5.0) for (a) tetrachlorocatechol and (b) trihexylamine, in POPC and POPC + 30% cholesterol.

As an initial investigation, we have calculated log *K*_MW_ for our full test set in lipid bilayers containing 30 mol % of cholesterol and compared this to our simulated values in pure phospholipid bilayers in Figure 10b. The impact of cholesterol in our Martini simulations varies according to the structure of the solute. For molecules containing rigid aromatic groups, cholesterol reduces membrane partitioning, while for small or flexible molecules, it is increased. Figure 11 illustrates the difference in free-energy profiles for tetrachlorocatechol and trihexylamine, which show these contrasting behaviors. Both profiles have a slightly lower free energy in the head-group region of the cholesterol-containing membrane, reflecting the lower concentration of phospholipid heads, but the partition coefficient is dominated by the free energy further into the membrane. Tetrachlorocatechol has a higher free energy in the tail region of the cholesterol membrane. This follows the pattern seen in many studies, where the increase in membrane stiffness on adding cholesterol hinders the reorganization of the lipids, which is required when a solute is added.^96,100−102^ In the case of trihexylamine, the free energy is lower in the tail region of the cholesterol membrane. This molecule is more flexible, reducing the lipid reorganization that is required on insertion, and so the favorable interactions with the denser membrane dominate the free-energy profile.

#### 3.3.3 Ionic Molecules

Many of the molecules studied in the previous sections have acidic or basic character, and their protonation state, and therefore charge, will change on moving through a lipid bilayer. We have modeled all of these molecules using neutral Martini beads. These beads are parameterized to match organic solvent–water partitioning data and therefore account for these changes in protonation state in an effective, implicit way.

Strongly ionic molecules are more difficult to model using Martini. The polarizable Martini model, which includes a water model with orientational polarizability, can help with some of the issues. However, the problem remains that there is only a limited selection of ionic bead types within Martini 2, which limits the ionic groups that can be represented. This means our mapping procedure is not guaranteed to work with all ionic molecules.

**Figure 12:**
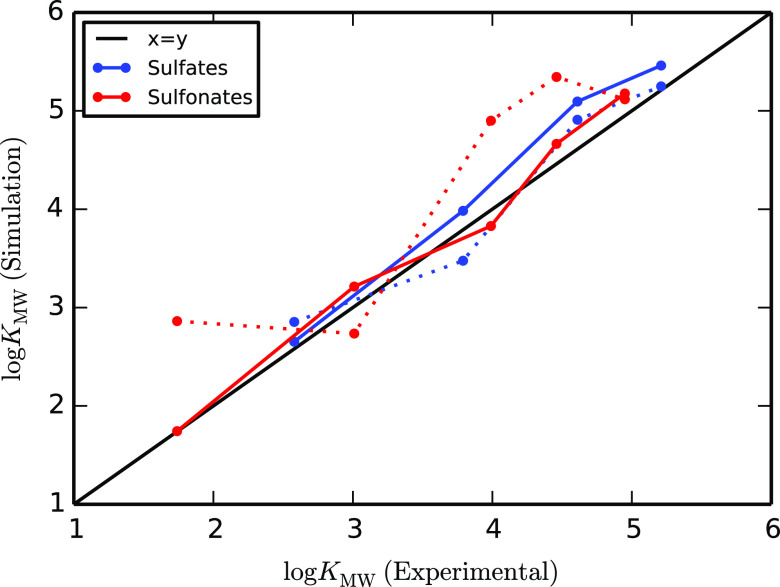
Membrane–water partition coefficients (log *K*_MW_) for alkyl sulfates (chain lengths 8, 10, 12, and 13) and sulfonates (chain lengths 8, 10, and 12–14), with models generated with predefined fragment matching (solid lines) and with the standard mapping procedure (dashed lines). In all cases, increasing the chain length increases log *K*_MW_.

With prior knowledge of which functional groups can be represented by particular charged bead types, it is possible to automatically generate models of molecules containing those groups. An additional preprocessing step is added to the mapping scheme where predefined fragments are mapped in a similar way to the aromatic patterns. The bead type for these mappings is also predefined. For example, the SO_4_^–^ and CSO_3_^–^ groups in organic sulfate and sulfonates are represented well by the Qa and Q0 bead types, respectively. This is shown in Figure 12, where these models and models generated without the additional preprocessing are compared to experimental data.^8^ Both sets of sulfate models give good results. For the sulfonates, the models based on predefined fragments give significantly improved results over the standard set of models.

Care is needed when sampling the free-energy profiles of ionic molecules. The force constant used in the restraint potential of the umbrella sampling for neutral molecules was weak enough that the charged group in the molecule could be pulled away from the desired depth and toward the charged region of the membrane. This behavior prevented convergence of the histograms from the two molecules. The problem was fixed by increasing the force constant from 1000 to 2000 kJ mol nm^–2^, and reducing the window spacing to 0.05 nm. For neutral molecules, the weaker force constant was sufficient to obtain converged histograms due to the absence of electrostatic interactions between the solute and the membrane. For those molecules, the increased computational expense of narrower umbrella windows brings no significant improvement in accuracy and so the weaker force constant is preferable.

Predefined mapping for ionic groups can give improved results across specific series of ionic surfactants. However, it is still limited by the resolution of Martini and knowledge of appropriate bead assignments. This is one area where the increased diversity of bead types in the new Martini 3 force field^75^ could open up the chemical space that can be covered by our automated approach.

## 4 Discussion

### 4.1 Scalability

**Figure 13:**
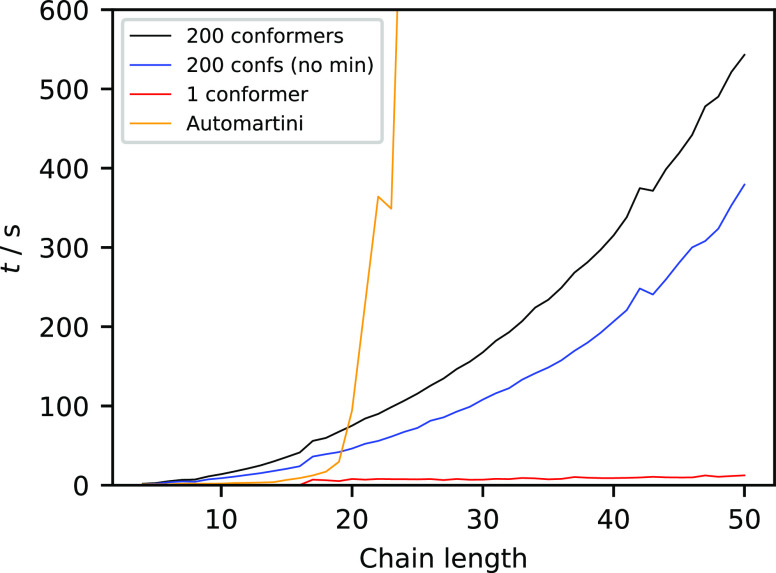
CPU time of the parameterization for linear alkanes of different chain lengths, with and without UFF minimization of the 200 atomistic conformers. Timings for automartini^22^ are also included for reference.

One advantage of the graph-based mapping algorithm is its computational speed. Even for large molecules, the division into beads takes a matter of seconds and so the approach scales very well with molecule size. The limiting factor for scalability of the coarse-graining procedure as a whole is the conformer generation required for parameterizing bonded interactions. As shown in Figure 13, the entire process for linear alkanes with just one conformer takes only seconds even for C_50_. When 200 conformers (the default in our code) are used, the computational time becomes noticeable. This reflects the cost of generating the conformers using ETKDG and looping over them to calculate their dimensions. Even so, the cost is still small compared to the simulations that are then carried out using the model, so the creation of the CG model would not be the limiting factor in predicting partition coefficients. For reference, on a cluster with 64 GB of RAM, the automartini code^22,57^ took 3.7 h to parameterize a C_29_ molecule, and C_30_ could not be parameterized due to memory constraints. With our method, the same molecules took 155 and 167 s, respectively.

While the mapping algorithm is, in principle, scalable to very high molecular weights, the low availability of experimental log *K*_MW_ for very large molecules does limit the sizes that can be included in our data set, which range from 2 to 28 heavy atoms. Benchmarking of much larger molecules would therefore require a different application from membrane–water partitioning to be chosen.

Our coarse-graining method relies on a principle of additivity, i.e., that a molecule can be constructed by joining beads that have been parameterized to represent isolated fragments.^22^ It is possible for the most appropriate Martini bead type for a fragment to depend on its chemical surroundings (as demonstrated, for example, in peptides^103^) and the errors associated with this dependence may accumulate as the molecular weight increases. Further work on ways to account for a fragment’s environment when choosing a bead type will be extremely valuable and in combination with our mapping approach will further extend the chemical space for which Martini can be used in high-throughput screening studies.

### 4.2 Nonbonded Parameters

Martini 2 was used in this paper because it is by far the most widely used coarse-grained force field. However, the mapping stage of our scheme is suitable for any force field with the same coarse-grained resolution, such as the recently released Martini 3 model, or any future coarse-grained models. Full automation of the process for new force fields will likely require new approaches to selecting bead types in the parameterization stage. Martini 3, for example, has more bead sizes and more hydrogen-bonding bead types than Martini 2,^66,75^ and differentiating between fragments based only on Δ*G*_OW_ will not be sufficient. Here, we have concentrated on Martini 2 as the simplest version of the force field. However, extension to new force fields can further improve the chemical space coverage of automated coarse-graining, particularly for charged molecules.

The intended bead size in Martini 2 is 4–5 heavy atoms but some of our mappings contain sizes outside this range. The criterion we used for bead size was a compromise between the preferred Martini size and the need for the method to work for any molecule. This includes not only linear or ring fragments but also highly branched structures. In the most branched molecules, such as perfluorinated alkyl chains, beads of 6–8 heavy atoms are possible. In these extreme cases, the larger beads are smaller in radius than a linear bead representing the same number of atoms and help to avoid excessively short bond lengths between beads. Nevertheless, the variation in bead size may cause artifacts in the free-energy profile for molecules where many beads are outside the 4–5 heavy atom range for which the beads were parameterized,^104^ as well as affecting other properties. Martini 2 does have small bead types, but these are identical to the normal bead types aside from their scaled interaction with other small beads.^49^ Extension to Martini 3 may alleviate these issues, as the newer force field has more sophisticated treatment of small bead types.^75^

The models generated using our method are intended for use in screening for thermodynamic properties. However, they may also be used as a starting point for a more detailed parameterization. For example, bead types may need to be fine-tuned if the automated assignment is not appropriate. This can occur when the behavior of an individual fragment differs significantly from the fragment within a larger molecule.^103^ For example, if an ester linkage (-C(=O)O-) is assigned to a bead by the mapping algorithm, the addition of the terminal hydrogen atoms will give formic acid, which is chemically very different. In that example, we found that a P3 bead reproduced partition coefficients better than the automatically assigned P1 bead.

### 4.3 Bonded Parameters

The bonded parameters in our models are sufficient for calculating thermodynamic properties. However, bulk and structural properties require a change of priority when parameterizing intramolecular interactions. Adjusting the automated bonded interactions based on molecule size rather than center-of-mass distances between beads should improve the accuracy of the model for properties such as bulk density, particularly in combination with Martini 3 bead types.^75^ However, it is important to note that even with improved bonded interactions, coarse-grained models based on pair potentials will be limited in their ability to correctly represent all of the structural and thermodynamic properties of the underlying atomistic system simultaneously.^30,105^ While it is desirable for a model to represent the real system as closely as possible, the simplicity of the model will always come with trade-offs in the properties that can be matched.

All of the automatically generated models shown in this paper have been tested and found to produce a stable simulation with umbrella sampling and a time step of 20 fs, which is a similar level of performance to handmade models of similar systems.^59^ For even more complex aromatic molecules with many substituents, a shorter time step may sometimes be necessary since keeping the correct geometry of such molecules will require many bonded parameters acting on a small number of beads.

## 5 Conclusions

We have developed an efficient, automated coarse-grained mapping method that is compatible with Martini and other force fields of a similar resolution. The method consists of graph-based mapping for nonring fragments and pattern-matching for ring fragments. Using this approach, we can generate models for a large number of organic molecules. This includes a wide variety of functional groups, as well as linear, branched, and ring compounds. The resulting models reproduce both octanol–water and membrane–water partition coefficients of the solute molecules well. Compared to previous approaches, we have expanded the range of ring-containing molecules that are accessible by introducing a new method of parameterizing virtual sites and constraints. The structures are stable even with a 20 fs time step, which is generally difficult to achieve for this type of molecule.

The new mapping and parameterization approach paves the way for routine use of coarse-grained modeling in high-throughput screening studies involving molecules of industrial interest. Due to the large chemical space that can be covered, it has the potential to be used as an alternative to simpler screening approaches that make more assumptions. We have demonstrated this point for simple lipid bilayers, including preliminary results for membranes that contain cholesterol, but the approach should be applicable to screening for partitioning into other systems for which there is a well-characterized Martini model. Further refinements of the method, including extension to Martini 3, will continue to expand the accessible chemical space and open up further applications.

## Supporting Information Available

Additional details of the parameterization of virtual sites and constraint networks for ring compounds, and results on the effects of using different bond parameterization schemes (PDF)
Full implementation of the algorithm for generating coarse-grained models described in this study (ZIP)
Gromacs topologies for all of the coarse-grained models generated in the study (ZIP)
Data file containing a full list of compounds studied, along with their experimental and simulated partitioning data (XLSX)
