# Supplementary material for: Automated Coarse-Grained Mapping Algorithm for the Martini Force Field and Benchmarks for Membrane–Water Partitioning
Source: J Chem Theory Comput. 2021 Sep 2;17(9):5777–91. doi: 10.1021/acs.jctc.1c00322 (PMC8444346; doi:10.1021/acs.jctc.1c00322)
Supplement: Supplementary file 1 — ct1c00322_si_001.pdf [file ct1c00322_si_001.pdf]

Supporting Information for An automated coarse-grained mapping algorithm for the Martini force field and benchmarks for membrane–water partitioning

Thomas D. Potter,† Elin L. Barrett,‡ and Mark A. Miller*,†

†*Department of Chemistry, Durham University, South Road, Durham DH1 3LE, United Kingdom*

‡*Unilever Safety and Environmental Assurance Centre, Colworth Science Park, Sharnbrook, Bedfordshire MK44 1LQ, United Kingdom*

E-mail: m.a.miller@durham.ac.uk

Contents

1 Derivation of virtual site equations 2

2 Procedure for generating ring geometries 3

3 Effect of bonded parameters 4

References 6

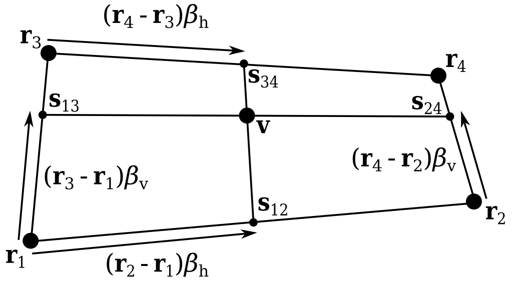

Figure 1: Schematic of the geometric constructions used to determine weights for a virtual site with $n = 4$ neighbours.

1 Derivation of virtual site equations

The equations which are solved to calculate the weights for virtual sites are given in Section 2.3.2. The derivation for these equations in the case where the virtual site has $n = 4$ neighbours is given here. The endpoints of the lines which cross the box, $\mathbf{s}_{ij}$, (see Figure 1) can be written in terms of the horizontal and vertical components of the site weights, β_h and β_v, and the positions of the real sites, $\mathbf{r}_i$. $\mathbf{r}_1$ is set to the origin for simplicity.

$$\begin{aligned}
\mathbf{s}_{12} &= \beta_\mathrm{h}\mathbf{r}_2, \\
\mathbf{s}_{13} &= \beta_\mathrm{v}\mathbf{r}_3, \\
\mathbf{s}_{24} &= \mathbf{r}_2 + \beta_\mathrm{v}(\mathbf{r}_4 - \mathbf{r}_2), \\
\mathbf{s}_{34} &= \mathbf{r}_3 + \beta_\mathrm{h}(\mathbf{r}_4 - \mathbf{r}_3).
\end{aligned} \tag{1}$$

The position of the virtual site, $\mathbf{v}$, is:

$$\begin{aligned}
\mathbf{v} &= \mathbf{s}_{12} + \beta_\mathrm{v}(\mathbf{s}_{34} - \mathbf{s}_{12}) \\
&= \beta_\mathrm{h}\mathbf{r}_2 + \beta_\mathrm{v}[\mathbf{r}_3 + \beta_\mathrm{h}(\mathbf{r}_4 - \mathbf{r}_3 - \mathbf{r}_2)].
\end{aligned} \tag{2}$$

Substituting $\mathbf{t} = \mathbf{r}_4 - \mathbf{r}_3 - \mathbf{r}_2$, and separating into x and y components, we get two equations:

$$\beta_\mathrm{h} r_{2x} - v_x + \beta_\mathrm{v}(r_{3x} + \beta_\mathrm{h} t_x) = 0 \tag{3a}$$

$$\beta_\mathrm{h} r_{2y} - v_y + \beta_\mathrm{v}(r_{3y} + \beta_\mathrm{h} t_y) = 0. \tag{3b}$$

Making β_v the subject of Eq. (3a) gives

$$\beta_\mathrm{v} = \frac{v_x - \beta_\mathrm{h} r_{2x}}{r_{3x} + \beta_\mathrm{h} t_x} \tag{4}$$

and substituting into Eq. (3b) gives

$$(r_{2y}t_x - r_{2x}t_y)\beta_\mathrm{h}^2 + (r_{2y}r_{3x} - r_{2x}r_{3y} - v_y t_x + v_x t_y)\beta_\mathrm{h} + v_x r_{3y} - v_y r_{3x} = 0. \tag{5}$$

The substitutions in Eq. (6) of the main article yield the quadratic Eq. (5) for β_h in the main article. Eq. (4) above then provides the value of β_v.

2 Procedure for generating ring geometries

This section details the algorithm for generating the constraint and dihedral network described in Section 2.3.3. In the following pseudocode, the ring of real sites is indexed in clockwise order from 0 to n. For a set of beads $ijkl$, the algorithm generates constraints jk and dihedrals $ijkl$ within one loop. The algorithm stops once the total number of constraints, C, is reached. The indices j and k are iterated in opposite direction round the ring, starting from n and 1 respectively.

```
C=n-3
j=n; k=1
while constraints to add:
    i=(j+1) mod (n+1); l=k+1
    add constraint jk
    add dihedral ijkl
    if C constraints added:
        stop
    k=k+1
    i=k-1; l=j-1
    add constraint jk
    add dihedral ijkl
    j=j-1
```

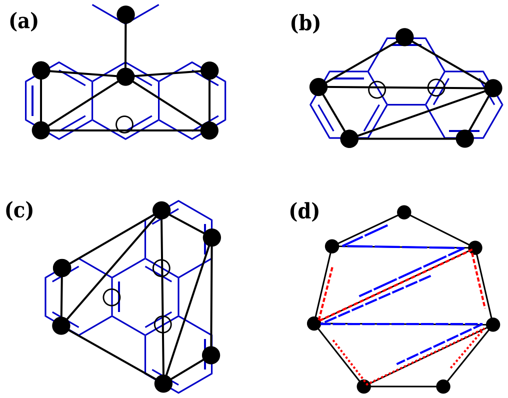

Figure 2: Coarse-grained ring geometries for (a) anthracene, (b) phenanthrene and (c) triphenylene. Each figure shows real sites as filled circles joined by constraints, and virtual sites as hollow circles which are not part of the constraint network. (d) A constraint and dihedral network for a seven-membered ring of real sites.

Figure 2(a)–(c) shows examples of ring structures with their coarse-grained beads and constraint structures overlaid. One limit of the automated method is the seven-membered ring of real sites illustrated in Fig. 2(d). At this size, the edges of dihedrals begin to overlap, and the automatically generated model becomes unstable during MD integration. However, it is rare to encounter this situation; most ring systems with up to four connected rings, and some larger, are represented by six or fewer real sites. In the case of even larger ring systems, the automatically generated mapping can act as a starting point for manual adjustment of the constraint and dihedral network, or reassignment of additional real sites to virtual sites.

3 Effect of bonded parameters

Figure 3 shows the convergence of bonded parameters for dodecane as the number of conformers generated, n, increases. Dodecane is modelled by three identical coarse-grained beads, so the two equilibrium bond lengths should be identical. As expected, very low numbers of conformers lead to inconsistent results. However, the average bond lengths and angles converge relatively quickly. When n reaches 200 there is a high level of confidence that the value calculated is close to the true average for the conformer generation method used. There are small differences between the two even at high n, but again, when n reaches 200

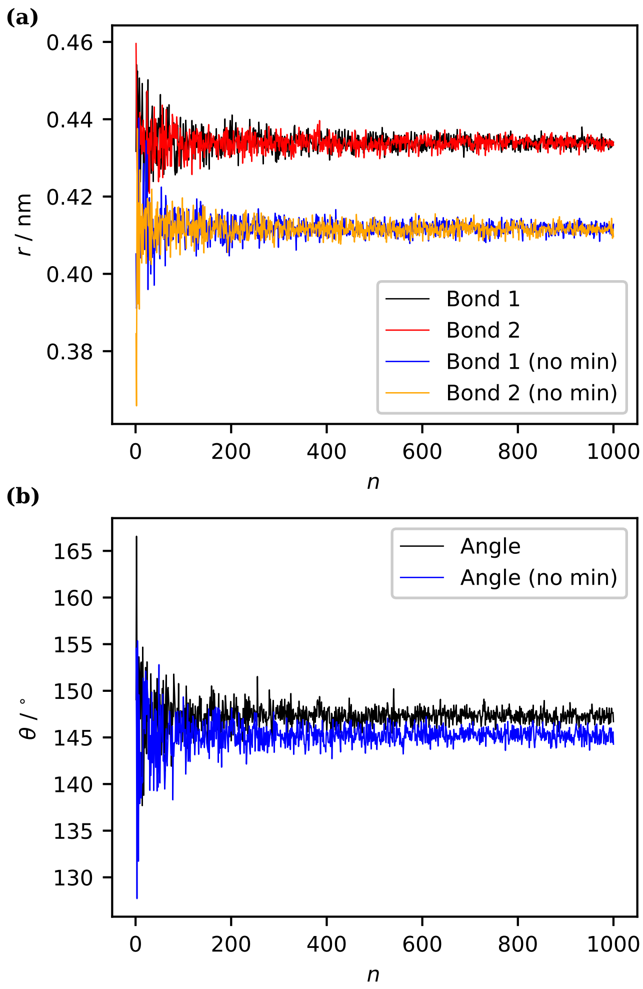

Figure 3: Convergence of coarse-grained (a) bond lengths and (b) bond angles of dodecane with the number of conformers, n, used to parametrise the interactions. Results are shown for conformers with and without UFF minimisation.

these differences are negligible.

The bonded parameters, particularly the bond lengths, changed significantly depending on whether the atomistic conformers from ETKDG were minimised. It should be noted that ETKDG conformers do not strictly require minimisation, as the method aims to directly predict crystal structures.[1] However, we investigated the influence of different conformer generation schemes by testing both dodecane models for density and partitioning. We compared these results to a model where the bonded parameters were generated using a more standard bottom-up procedure, in which a single-molecule atomistic simulation was run

Table 1: Experimental and simulated properties of dodecane using bond lengths (r) from different parametrisation schemes.

Model	r / nm	ρ / kg m^{-3}	logK_{OW}
ETKDG	0.415, 0.408	725	7.08
ETKDG + minimisation	0.434, 0.435	689	7.10
OPLS	0.458, 0.458	659	7.13
Standard Martini	0.47, 0.47	646	7.17
Experimental	—	750	6.10

using the OPLS force field.[2]

Unsurprisingly, the bonded parameters have a significant effect on the density. ETKDG-derived parameters give a density closer to the experimental value than those obtained after minimisation, or after an OPLS simulation. However, this does not necessarily mean that the latter methods are less accurate at determining bonded parameters. Density is affected by both bonded and non-bonded parameters, and so obtaining an accurate density requires fine-tuning of both. In contrast, logK_{OW} is not significantly affected by changing bond lengths, as the dominant factors here are the non-bonded interactions. If thermodynamic properties are of interest, therefore, the method used to parametrise bonded interactions is less important, and fine-tuning may not be necessary.

References

(1) Riniker, S.; Landrum, G. A. Better Informed Distance Geometry: Using What We Know To Improve Conformation Generation. *J. Chem. Inf. Model.* **2015**, *55*, 2562–2574.

(2) Dodda, L. S.; Cabeza de Vaca, I.; Tirado-Rives, J.; Jorgensen, W. L. LigParGen web server: an automatic OPLS-AA parameter generator for organic ligands. *Nucleic Acids Res.* **2017**, *45*, W331–W336.
