# Supplementary material for: Automated Coarse-Grained Mapping Algorithm for the Martini Force Field and Benchmarks for Membrane–Water Partitioning
Source: J Chem Theory Comput. 2021 Sep 2;17(9):5777–91. doi: 10.1021/acs.jctc.1c00322 (PMC8444346; doi:10.1021/acs.jctc.1c00322)
Supplement: Supplementary file 2 — ct1c00322_si_002.zip [file ct1c00322_si_002.zip › readme.pdf]

An automated coarse-grained mapping algorithm for the Martini force field and benchmarks for membrane–water partitioning

Thomas D. Potter,[1] Elin L. Barrett[2] and Mark A. Miller[1]

[1]Durham University, Department of Chemistry
[2]Unilever, Safety and Environmental Assurance Centre

This script can be used to automatically generate a coarse-grained model of an organic solute using the Martini forcefield parameters. The coarse-grained models are compatible with the Gromacs simulation package (recommended 2018 or newer). If you use this method, please cite the attached paper, DOI:10.1021/acs.jctc.1c00322.

The following files are included:

- *cg_param.py* – automatically generates a coarse-grained model of an organic solute
- *fragment_DGs.dat* – contains ΔG_{OW} values for organic fragments which could make up individual coarse-grained beads

The following external packages are required, all of which are freely available and open source:

- Python 3.7 or newer with the following packages:
  - numpy
  - scipy
  - RDKit, version 2020 or newer
  - requests

The easiest way to install the correct python packages is using the conda package manager, for example from: `https://docs.conda.io/en/latest/miniconda.html`. A working environment can be created using:

```
$ conda create -c rdkit -n cg_param rdkit numpy scipy requests
$ conda activate cg_param
```

The most recent version of Gromacs, and detailed installation instructions, can be found at: `https://manual.gromacs.org/documentation/current/download.html`.

The command used to parametrise, for example, an octane molecule would be:

```
$ cg_param.py "CCCCCCCC" octane.gro octane.itp
```
